# Opinion: Defining Readiness for Reconstructive Surgery in Patients With Advanced Pressure Ulcers—A Policy and Clinical Perspective

**DOI:** 10.1111/wrr.70099

**Published:** 2025-10-07

**Authors:** Alica Hokynková, Petr Šín, Dimitri Beeckman, Vladimír Váňa, Hana Paulová, Andrea Pokorná

**Affiliations:** ^1^ Department of Burns and Plastic Surgery University Hospital Brno Brno Czech Republic; ^2^ Faculty of Medicine Masaryk University Brno Czech Republic; ^3^ Faculty of Medicine and Health, School of Health Sciences, Nursing Science Unit, Swedish Centre for Skin and Wound Research (SCENTR) Örebro University Örebro Sweden; ^4^ Department of Public Health and Primary Care, University Centre for Nursing and Midwifery, Skin Integrity Research Group (SKINT) Ghent University Ghent Belgium; ^5^ Department of Biochemistry, Faculty of Medicine Masaryk University Brno Czech Republic; ^6^ Department of Health Sciences, Faculty of Medicine Masaryk University Brno Czech Republic

Dear Editor,

Reconstructive surgery for advanced pressure ulcers continues to pose one of the most persistent challenges in wound care. Despite advances in surgical techniques, flap coverage remains burdened by unacceptably high complication and recurrence rates, with devastating consequences for patients. In a large series, nearly 59% of patients experienced complications, including wound dehiscence in 31% and recurrence approaching 29% [1]. In spinal cord injury cohorts, dehiscence reached 31%, infection 25%, hematoma 20% and flap necrosis 10%–14% [2]. Even more concerning, long‐term follow‐up from a national database reported recurrence in 73.6% of patients [3].

These outcomes are not merely statistics; they represent repeated operations, prolonged hospital stays and significant losses in quality of life. For clinicians, they highlight a critical gap: the absence of clear, evidence‐based readiness criteria to determine when a patient is truly prepared for reconstruction.

International guidelines provide important broad nutritional recommendations, such as 30–35 kcal/kg/day and 1.25–1.5 g protein/kg/day, but do not define laboratory thresholds to guide surgical timing. Even in the new 2025 Quick Reference Guide from NPIAP/EPUAP/PPPIA, despite updated nutrition recommendations and good practice statements for prevention, there remains no specification of laboratory cut‐off values to determine surgical readiness [4]. The 2023 WHS update acknowledges that albumin and prealbumin are unreliable in hospitalised and inflamed patients [5]. ESPEN guidelines emphasise nutritional screening, optimisation and high‐protein diets for surgical patients, but again provide no explicit laboratory cut‐off values or “go/no‐go” criteria to determine readiness for surgery [6]. In practice, this leaves decisions to clinical judgement, inevitably leading to variation across centres.

To address this gap, our institution has introduced pragmatic internal readiness criteria (Table 1): haemoglobin > 100 g/L, CRP < 50 mg/L, total protein > 60 g/L, albumin > 30 g/L and prealbumin > 0.15 g/L. In patients colonised with multidrug‐resistant organisms, barrier precautions and targeted antibiotics are mandatory. These cut‐offs are grounded in clinical reasoning: anaemia impairs tissue oxygenation, elevated CRP reflects uncontrolled infection and low protein indices consistently correlate with flap dehiscence and failure [7, 8].

**TABLE 1:** Criteria for assessing readiness for elective flap reconstruction in pressure ulcer patients at the University Hospital Brno.

Parameter	Threshold
Haemoglobin	> 100 g/L
CRP	< 50 mg/L
Total protein	> 60 g/L
Albumin	> 30 g/L
Prealbumin	> 0.15 g/L
MDR colonisation	Barrier precautions and targeted antibiotics

Malnutrition is highly prevalent in the critically ill, with reported rates of up to nearly 80%. Importantly, many patients are already malnourished on hospital admission, and without targeted nutritional support, their condition often worsens during hospitalisation [9]. Exudative wounds further accelerate protein loss, which in more severe or infected wounds can reach a few grams per day—for example, up to ~2.1 g/day in older patients. Other reports suggest even higher losses in different wound types, such as burns or open abdomen [10]. Yet, these losses are rarely acknowledged in existing guidelines, placing patients at risk of significant underfeeding and poor surgical outcomes.

We propose that surgical timing should not rely solely on clinical impression. A minimal perioperative panel, monitored at least twice weekly, provides an objective foundation: CBC with haemoglobin, CRP, electrolytes and glucose, total protein, albumin, prealbumin, weight, BMI and dietitian assessment. If criteria are not met, elective reconstructive surgery should be delayed. In the case of a septic pressure ulcer, urgent surgical opening and source control are required; however, definitive flap reconstruction should proceed only once the predefined criteria are achieved.

We acknowledge the limitations: these criteria are institutionally defined and not yet validated in prospective trials. They arise from long‐term clinical practice rather than randomised evidence. However, this pragmatic approach highlights the need for dialogue. By sharing institutional thresholds, we hope to stimulate multicentre collaboration to compare practices, refine criteria and ultimately validate readiness measures for reconstructive surgery.

The stakes are high. Failure after flap surgery is devastating for patients and demoralising for surgical teams. Of course, there are also high costs associated with care. These may be questioned precisely because of the failure. Timing matters. Integrating metabolic, nutritional and inflammatory readiness into reconstructive planning offers a pathway to safer, more standardised care. Establishing and validating practical readiness criteria should be a priority for the wound care and surgical community.

## Conflicts of Interest

The authors declare no conflicts of interest.

## Data Availability

Data sharing is not applicable to this article as no datasets were generated or analysed during the current study.
